# Supplementary material for: NMR Characterization of Long-Chain Fatty Acylcarnitine Binding to the Mitochondrial Carnitine/Acylcarnitine Carrier
Source: Int J Mol Sci. 2022 Apr 21;23(9):4608. doi: 10.3390/ijms23094608 (PMC9103206; doi:10.3390/ijms23094608)
Supplement: Supplementary file 1 [file ijms-23-04608-s001.zip › ijms-1651531-supplementary.pdf]

International Journal of
Molecular Sciences

MDPI

Article

NMR Characterization of Long-Chain Fatty Acylcarnitine Binding to the Mitochondrial Carnitine/Acylcarnitine Carrier

Ningning Zhang [1,2], Xiaopu Jia [3], Shuai Fan [3], Bin Wu [4], Shuqing Wang [3,*] and Bo OuYang [1,2,*]

1 State Key Laboratory of Molecular Biology, Shanghai Institute of Biochemistry and Cell Biology, Center for Excellence in Molecular Cell Science, Chinese Academy of Sciences, Shanghai 200031, China; zhangningning2014@sibcb.ac.cn
2 University of Chinese Academy of Sciences, Beijing 100049, China
3 School of Pharmacy, Tianjin Medical University, Tianjin 300070, China; 20216020604@tmu.edu.cn (X.J.); 20216020602@tmu.edu.cn (S.F.)
4 National Facility for Protein Science in Shanghai, ZhangJiang Laboratory, Shanghai Advanced Research Institute, Chinese Academy of Sciences, Shanghai 201203, China; bin.wu@sibcb.ac.cn
* Correspondence: wangshuqing@tmu.edu.cn (S.W.); ouyang@sibcb.ac.cn (B.O.); Tel.: +86-021-54920143 (B.O.)

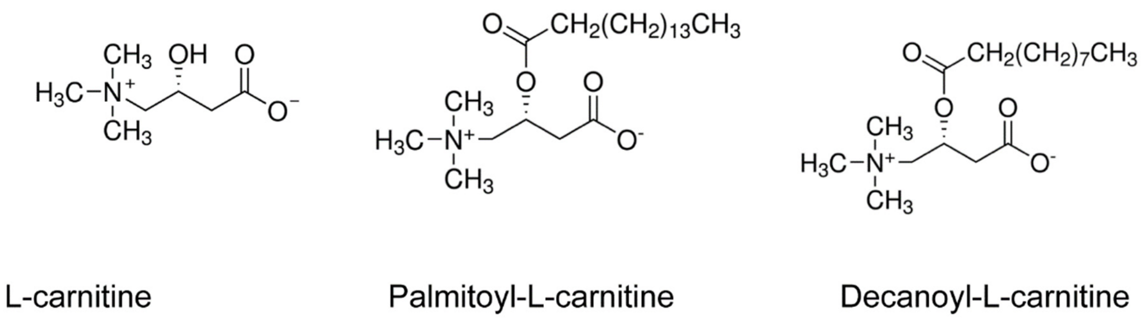

Figure S1. Chemical structures of L-carnitine, palmitoyl-L-carnitine and decanoyl-L-carnitine

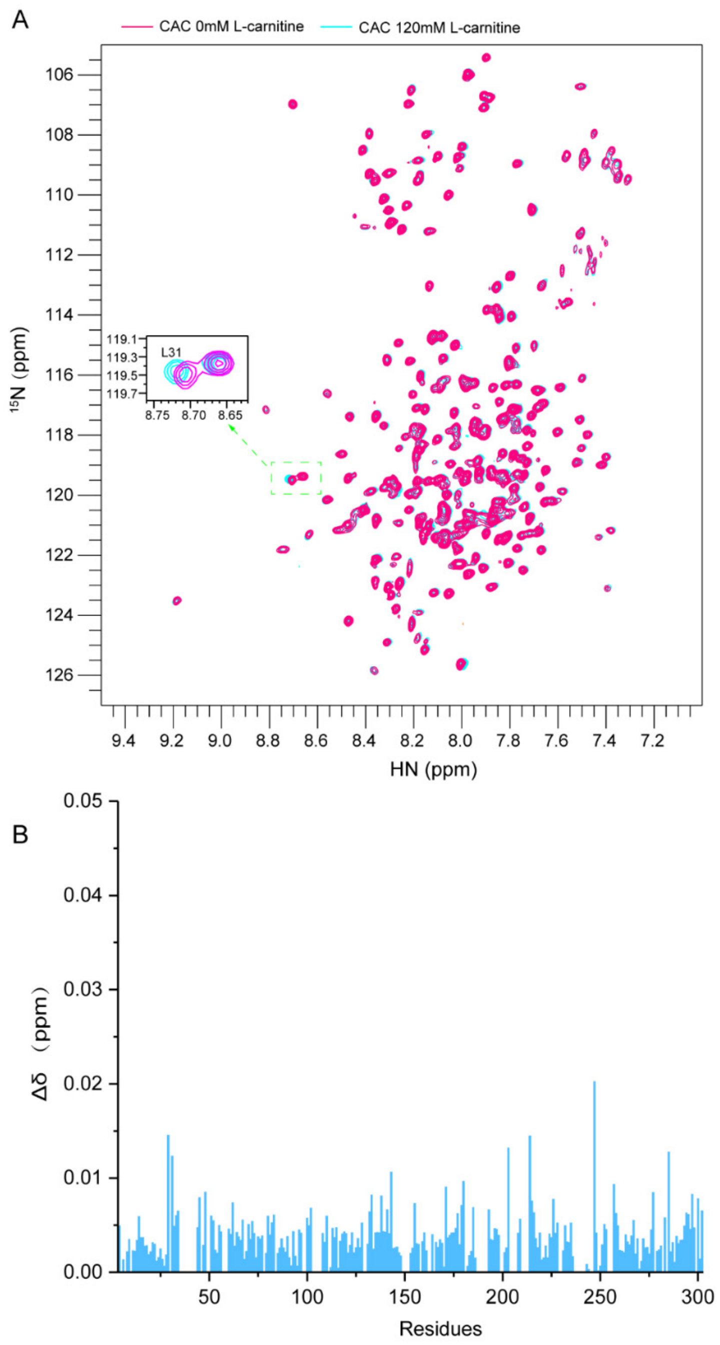

Figure S2. NMR titrations of L-carnitine into CAC in DPC micelles. (**A**) Superimposed 2D ^{1}H-^{15}N TROSY-HSQC spectra with (cyan) and without (pink) 120 mM L-carnitine; (**B**) The residue-specific chemical shift changes of the 2D ^{1}H-^{15}N TROSY-HSQC spectra between CAC with and without 120 mM L-carnitine.

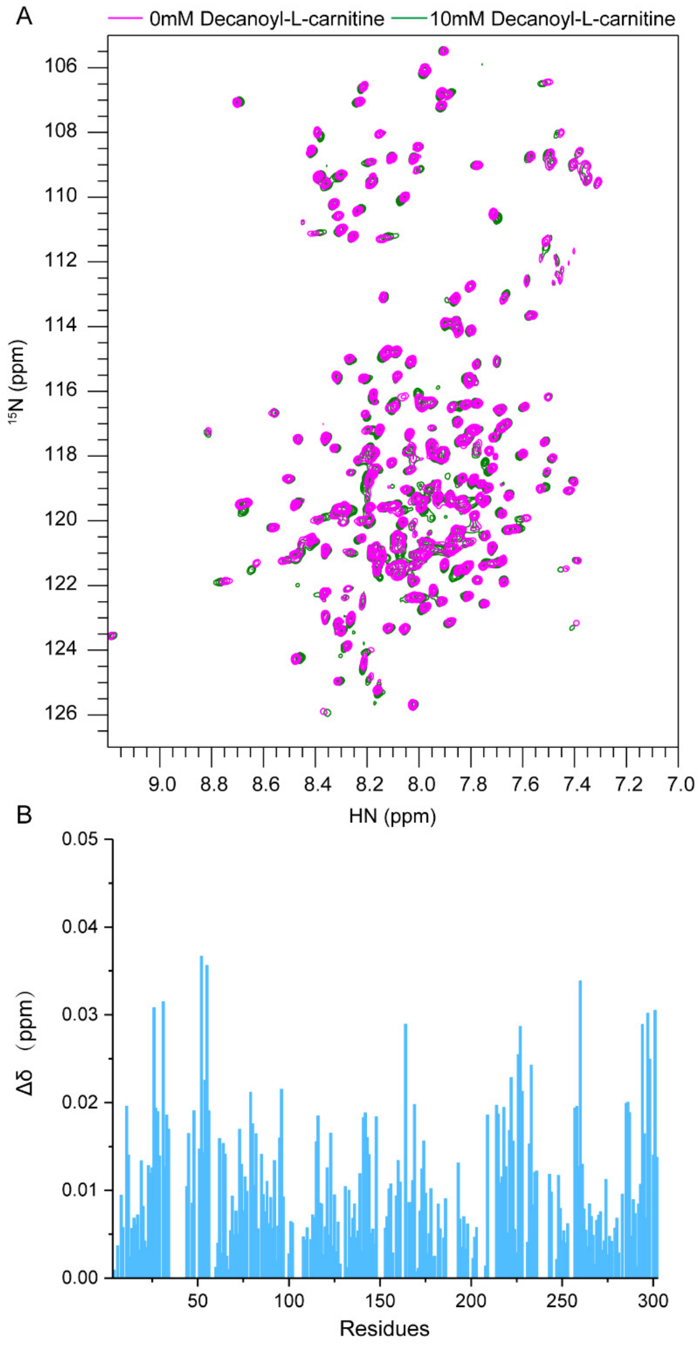

Figure S3. NMR titrations of decanoyl-L-carnitine into CAC. (**A**) Superimposed 2D ^{1}H-^{15}N TROSY-HSQC spectra with (green) and without (magenta) 10 mM decanoyl-L-carnitine; (**B**) The residue-specific chemical shift changes of the 2D ^{1}H-^{15}N TROSY-HSQC spectra between CAC with and without 10 mM decanoyl-L-carnitine.

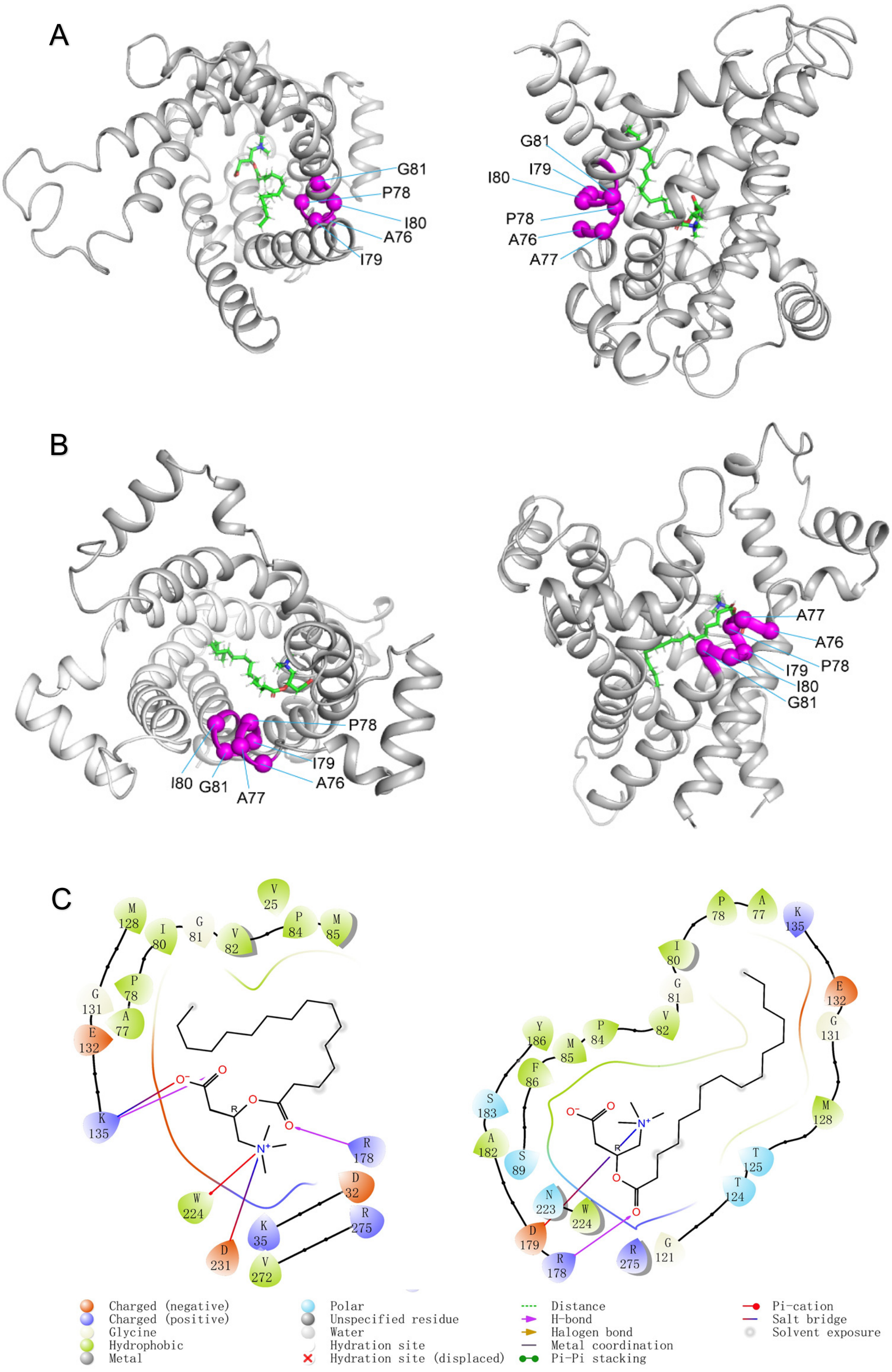

Figure S4. The two CAC models with palmitoyl-L-carnitine at the initial binding site. The two CAC homology models were generated using the AAC crystal structures of 1OKC (**A**) and 6GCI (**B**). Palmitoyl-L-carnitine (green) is the substrate binding to the model docked by AutoDock Vina1.1.2. (**C**) The type of interactions observed between the substrate and CAC, including region A76-G81 from NMR study (Left is c-state, right is m-state).

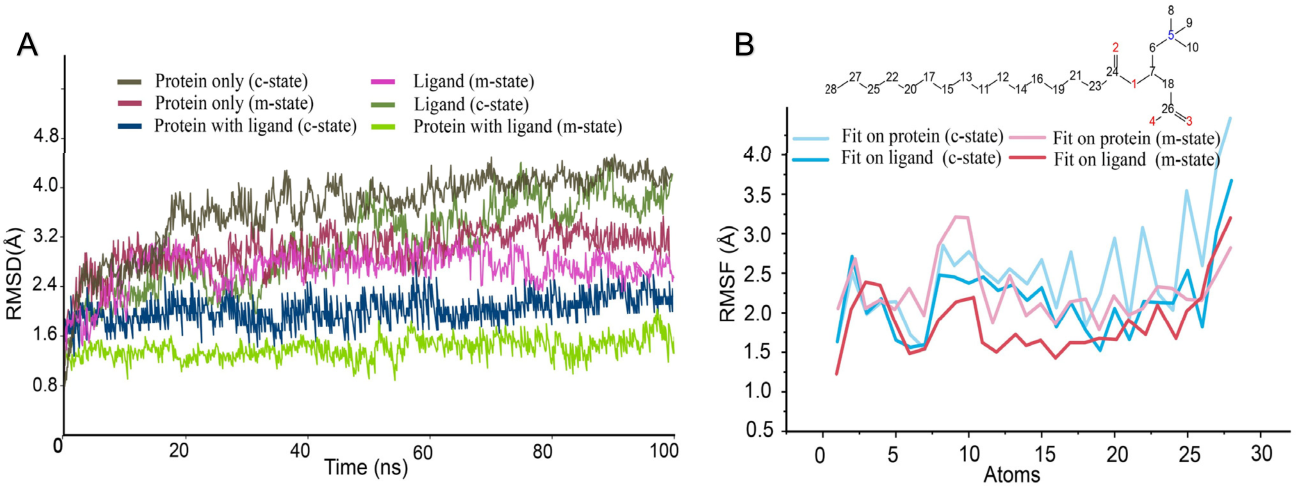

Figure S5. RMSD and RMSF plots of the CAC and palmitoyl-L-carnitine complex. (**A**) RMSD plots of the CAC and palmitoyl-L-carnitine complexes against simulation time, showing system convergence and stability of simulations. Olive lines: protein only for the c-state open apo-CAC; Purple lines: protein only for the m-state open apo-CAC; Green lines: palmitoyl-L-carnitine in the c-state open complex; Magenta lines: palmitoyl-L-carnitine in the m-state open complex; Blue lines: CAC protein in the c-state open complex; Light green lines: CAC protein in the m-state open complex. (**B**) RMSF plots of all atoms of palmitoyl-L-carnitine from the complexes. Light blue lines: Fluctuation of palmitoyl-L-carnitine atoms compared to protein CAC coordinates in c-state open complex; Blue lines: Fluctuation of palmitoyl-L-carnitine atoms compared to its own coordinates in the c-state open complex; Pink lines: Fluctuation of palmitoyl-L-carnitine atoms compared to protein CAC coordinates in the m-state open complex; Red lines: Fluctuation of palmitoyl-L-carnitine atoms compared to its own coordinates in the m-state open complex.

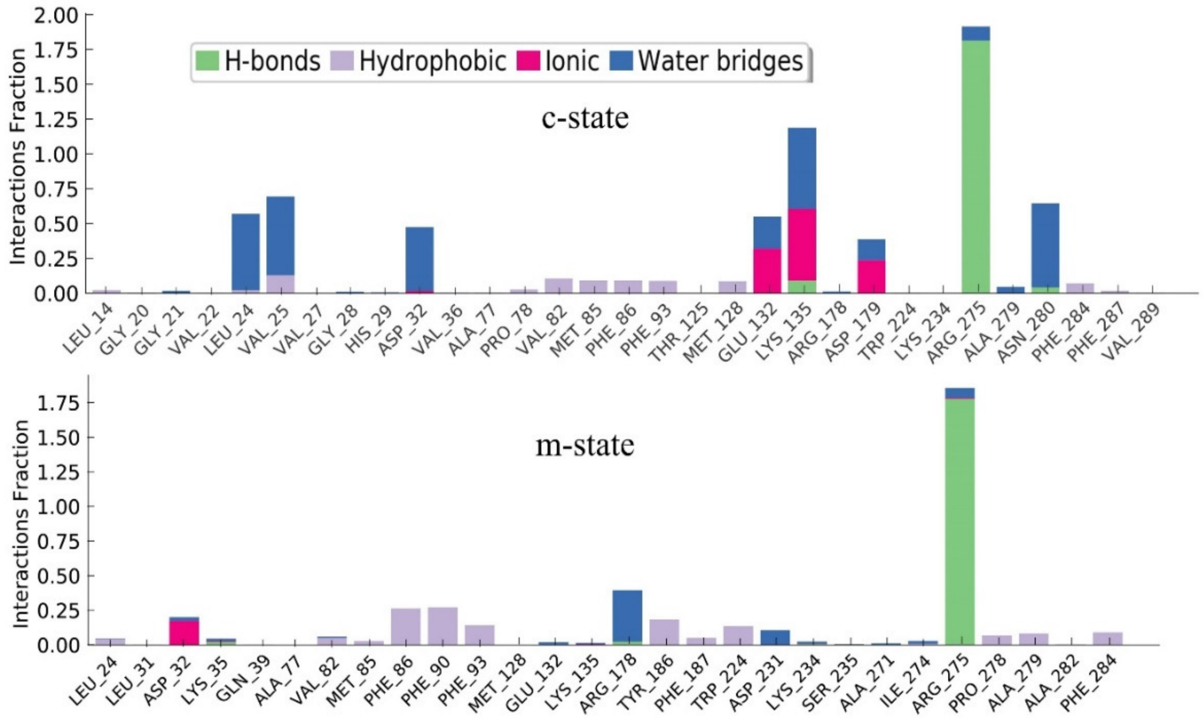

Figure S6. Protein interactions with the ligand are monitored throughout the simulation. These interactions can be categorized by type and summarized, as shown in the plot above. Protein-ligand interactions (or 'contacts') are categorized into four types: Hydrogen bonds, Hydrophobic, Ionic and Water bridges. The stacked bar charts are normalized over the course of the trajectory. The values of interaction fraction suggest that the percentage of the simulation time the specific interaction is maintained.

Table S1. The RMSD values of structure alignment of the protein backbones for different conformations.

	1OKC	Ci	Cf	6GCI	Mi	Mf
1OKC	0	1.512	3.365	4.162	3.928	3.653
Ci	1.512	0	0.543	4.040	4.208	4.219
Cf	3.365	0.543	0	4.232	4.308	3.750
6GCI	4.162	4.040	4.232	0	1.547	3.435
Mi	3.928	4.208	4.308	1.547	0	0.427
Mf	3.653	4.219	3.750	3.435	0.427	0

.
